# The Red Queen in mitochondria: cyto-nuclear co-evolution, hybrid breakdown and human disease

**DOI:** 10.3389/fgene.2015.00187

**Published:** 2015-05-19

**Authors:** Jui-Yu Chou, Jun-Yi Leu

**Affiliations:** ^1^Department of Biology, National Changhua University of Education, Changhua, Taiwan; ^2^Institute of Molecular Biology, Academia Sinica, Taipei, Taiwan

**Keywords:** cyto-nuclear incompatibility, Muller’s ratchet, selfish mitochondrial DNA, Red Queen effect, mitochondrial disease

## Abstract

Cyto-nuclear incompatibility, a specific form of Dobzhansky-Muller incompatibility caused by incompatible alleles between mitochondrial and nuclear genomes, has been suggested to play a critical role during speciation. Several features of the mitochondrial genome (mtDNA), including high mutation rate, dynamic genomic structure, and uniparental inheritance, make mtDNA more likely to accumulate mutations in the population. Once mtDNA has changed, the nuclear genome needs to play catch-up due to the intimate interactions between these two genomes. In two populations, if cyto-nuclear co-evolution is driven in different directions, it may eventually lead to hybrid incompatibility. Although cyto-nuclear incompatibility has been observed in a wide range of organisms, it remains unclear what type of mutations drives the co-evolution. Currently, evidence supporting adaptive mutations in mtDNA remains limited. On the other hand, it has been known that some mutations allow mtDNA to propagate more efficiently but compromise the host fitness (described as selfish mtDNA). Arms races between such selfish mtDNA and host nuclear genomes can accelerate cyto-nuclear co-evolution and lead to a phenomenon called the Red Queen Effect. Here, we discuss how the Red Queen Effect may contribute to the frequent observation of cyto-nuclear incompatibility and be the underlying driving force of some human mitochondrial diseases.

## Introduction

Mitochondria are the major energy source of the cell and are also involved in many important cellular functions (McBride et al., 2006). Although mitochondria harbor their own genome (mtDNA), mitochondrial genomes are severely degenerated and comprise a few to dozens of protein-coding genes, while the majority have been transferred to the host nuclear genome (Burger et al., 2003). To make functional mitochondria, hundreds of nucleus-encoded proteins are required (Meisinger et al., 2008). This close relationship between mitochondria and the host cell guarantees tight co-evolution between their genomes, which also implies that any mismatch may result in severe defects in cellular fitness.

According to the biological species concept, evolution of reproductive isolating barriers limiting gene flow between two populations represents a critical step of speciation (Coyne and Orr, 2004). Reproductive isolation can result from various molecular mechanisms, including anti-recombination induced by DNA sequence divergence, chromosome translocation and genetic incompatibility (Noor and Feder, 2006; Greig, 2009). In recent years, much attention has been paid to the identification of incompatible genes and various types of genetic incompatibility have been revealed to cause hybrid inviability or sterility (Wolf et al., 2010). Among them, cyto-nuclear incompatibility is the one that has been repeatedly observed between species or populations in fungi, plants and animals (Gershoni et al., 2009; Chou and Leu, 2010; Burton et al., 2013). Why does cyto-nuclear incompatibility occur so frequently across different kingdoms? Is there any specific driving force underlying the evolution of cyto-nuclear incompatibility?

At least three types of driving forces have been speculated to cause cyto-nuclear incompatibility: (a) adaptive divergence, (b) compensatory coadaptation, and (c) intergenomic conflict. One can distinguish adaptive divergence from the other two because the adaptive mutations were often fixed by selection from specific ecological environments. Using hybrid or cybrid (cytoplasmic hybrid which contains a nuclear genome from one source and cytoplasmic genomes from another) cells, the fitness of alternative organelles in different genomic and ecological environments can be measured to elucidate the role of extrinsic selection in intergenomic incompatibilities. In the case of compensatory coadaptation and intergenomic conflict, we expect to observe only deleterious or neutral effects from the mitochondrial genome. However, such mitochondrial genomes are able to spread through a naïve population at a rate higher than genetic drift under the scenario of intergenomic conflict (discussed further below).

Several incompatible cyto-nuclear gene pairs have been dissected to the molecular level (Harrison and Burton, 2006; Wang et al., 2006; Lee et al., 2008; Chou et al., 2010). Moreover, many studies have provided indirect evidence for the driving force underlying evolution of cyto-nuclear incompatibility in different organisms (Burton et al., 2013). In this review, we will discuss the possibility that the internal evolutionary arms race between mitochondrial and nuclear genomes accelerates co-evolution and causes hybrid incompatibilities, as well as the implication of cyto-nuclear incompatibility in human disease.

## Adaptive Divergence in Mitochondrial DNA

Some indirect evidence has suggested that mutations in mtDNA can be fixed due to adaptive evolution in the parental species. In two sunflower species, *Helianthus annuus* and *Helianthus petiolaris*, ecological selection is speculated to contribute to cyto-nuclear incompatibilities in the hybrid. Mismatches between mitochondrial and nuclear genomes significantly influenced the fitness and architecture of hybrid plants (Sambatti et al., 2008). Adaptive divergence in mtDNA has also been reported in animal studies. Ballard compared 22 *Drosophila simulans* and 2 *D. melanogaster* mitochondrial genomes and found an excess of non-synonymous substitutions relative to synonymous substitutions within each *D. simulans* mtDNA haplotype, indicating differential selection in the subdivided populations (Ballard, 2000). In a later study by James and Ballard, they tested three life-history traits on *D. simulans* strains that carried different mtDNA in a similar nuclear background. Significant differences in both development time and survivorship were observed among flies carrying distinct mitochondrial genotypes. The authors suggested that the changes in mtDNA might be driven by the infected Wolbachia strain in different geographic populations (James and Ballard, 2003). In a study using the seed beetle *Callosobruchus maculatus*, the authors observed complicated mtDNA-nDNA-environment interactions and speculated that thermal adaptation might be involved in this phenomenon (Arnqvist et al., 2010). Taken together, these studies suggest that ecological selection can contribute to the changes in mitochondrial DNA that may further lead to cyto-nuclear incompatibility between populations or species (Galtier et al., 2009). However, it remains unclear whether the adaptive mutations first occur in mitochondrial or nuclear genomes. It will require identification of the incompatible genes in order to address this question.

## Muller’s Ratchet in Mitochondria

Muller’s ratchet is the process by which the genome of an asexual population accumulates deleterious mutations in an irreversible manner. It is contrasting to what happens in a sexual population that deleterious mutations are able to be purged by genetic recombination. Consequently, the genetic load of an asexual population will become so great that the population may go extinct. The “ratchet effect” can also occur in the organelle genomes that do not recombine. Although mitochondrial and nuclear genomes coexist in the same cell, they often show different evolutionary trajectories. Several features of the mitochondrion make its genome more susceptible to Muller’s ratchet and genome degradation.

First, mitochondria are often inherited uniparentally and seldom undergo DNA recombination, which will influence their effective population size and also the ability to purge deleterious mutations. Many isogamous organisms have been observed to actively degrade paternal mtDNA, even though gametes from both parents contribute mtDNA to the zygote (Beckers et al., 1991; Sutovsky et al., 1999, 2000; Nakamura, 2010). In anisogamous organisms, mtDNA is predominantly inherited from the maternal parent, probably due to the size difference between male and female gametes. Thus, even in a sexual population, mitochondrial lineages are effectively asexual and more vulnerable to the “ratchet effect.”

Second, the mitochondrial genome is more susceptible to point mutations, deletions/insertions, and structural changes compared with the nuclear genome. DNA repair machinery in mitochondria is inefficient and mtDNA is not protected by histones or DNA-binding proteins (Avise, 1991; Shigenaga et al., 1994; Wei, 1998; Krishnan et al., 2008; Fukui and Moraes, 2009). In addition, the proximity to the electron transport chain may make mtDNA vulnerable to oxidative damage mediated by reactive oxygen species (Finkel and Holbrook, 2000). In yeast, genome size, gene order, and non-coding regions of mtDNA vary widely between different lineages (Solieri, 2010). In plants, although the nucleotide substitution rate of mtDNA is generally lower than nuclear DNA, mtDNA undergoes frequent recombination and its structure is highly dynamic (Wolfe et al., 1987; Palmer and Herbon, 1988).

Finally, it is worth noting that mtDNA often experiences small bottlenecks even though most cells contain dozens to thousands of mitochondrial genomes. In mammals, the genetic bottleneck may result from replication of a subpopulation of mtDNA (Wai et al., 2008) or a sharp reduction in the mtDNA copy number during embryogenesis (Cree et al., 2008). In yeast cells, it has been shown that mtDNA partitioning during vegetative segregation is non-random, which increases the rate of intracellular drift (Birky, 2001). When deleterious mutations accumulate in mtDNA, they inevitably compromise the fitness of host cells. Such a population may often go extinct when facing competition from other populations. However, if the population is well isolated, compensatory mutations in the nuclear genome will be selected to restore fitness.

## Selfish Mitochondrial DNA

Selfish DNA often spreads through a population without contributing adaptive benefits. On some occasion, the selfish element may be deleterious to the reproductive success of the host. One such example is the selfish mtDNA in the suppressive petite mutants discovered in yeast (Ephrussi et al., 1955; Hurst and Werren, 2001; Petersen et al., 2002). In some petite mutants, small mtDNA is able to replicate more efficiently than wild-type mtDNA and therefore has a higher chance to be transmitted to daughter cells (MacAlpine et al., 2001). The resulting petite cells are unable to respire and have a slower growth rate. Using laboratory evolution experiments, Jasmin and Zeyl (2014) showed that petite mutants could indeed spread in outcrossing sexual populations originally composed of respiration-competent cells. Some other fungal species also contain mitochondrial plasmids, which arise from rearrangements within the mitochondrial genome and over-replicate at the expense of wild-type mtDNA (Bertrand et al., 1985). Over-replication of dysfunctional mitochondrial genomes can lead to cell death and senescence (Griffiths, 1992). Recently, selfish mtDNA has been discovered in the nematode *Caenorhabditis briggsae* (Clark et al., 2012). The mtDNA in natural populations carries large heteroplasmic deletions, and some deletions exhibit a transmission bias. The selfish mtDNA produces damaging reactive oxygen species and influences the fecundity and pharyngeal pumping rates of the nematode. Transmission biases of small mtDNA are observed in flies and crickets as well. However, the impacts of small mtDNA on host fitness remain unclear (Solignac et al., 1984; Rand and Harrison, 1986).

Mitochondrial genomes are maternally inherited through the cytoplasm in many organisms. Thus, any mutations in mtDNA that increase female fitness will be selected for, even if they are deleterious to males. This inter-genomic conflict has well known associations with cytoplasmic male sterility (CMS) in plants (Fishman and Willis, 2006; Chase, 2007; Chen and Liu, 2014). CMS is a maternally inherited trait often associated with the appearance of chimeric genetic units in the mitochondrial genome, that is thought to result from aberrant recombination events (Hanson and Bentolila, 2004; Case and Willis, 2008). In many cases, a nuclear-encoded fertility restorer gene (*Rf*) was observed to restore fertility of the CMS plants (Barr and Fishman, 2010; Luo et al., 2013). Therefore, the CMS/*Rf* system also provides a valuable model for the study of interactions between nuclear and mitochondrial genomes (Horn et al., 2014; Hu et al., 2014). Interestingly, most *RF* alleles have been shown to belong to the pentatricopeptide repeat (PPR) protein family (Chase, 2007; Schmitz-Linneweber and Small, 2008). PPR proteins are often involved in post-transcriptional processes, such as RNA editing, RNA cleavage, and activation or repression of RNA translation (Barkan and Small, 2014). The *RF* alleles probably function in preventing the expression of unwanted or abnormal proteins generated from aberrant rearrangements of the organelle genome.

One important difference between selfish mtDNA and other selfish elements is that mtDNA propagates asexually (without recombination in most cases). All mutations in the same genome will hitchhike with the selfish mtDNA when it is spreading through the population. As discussed above, mitochondrial genomes are more susceptible to mutations than nuclear genomes. This implies that selfish mtDNA may have a high chance to carry other deleterious mutations even though the mutations causing selfish behavior are neutral to the host fitness. Consequently, the effect of compensatory coadaptation and intergenomic conflict may occur on the same mitochondrial DNA.

## Co-evolution between the Nuclear and Mitochondrial Genomes

**FIGURE 1:**
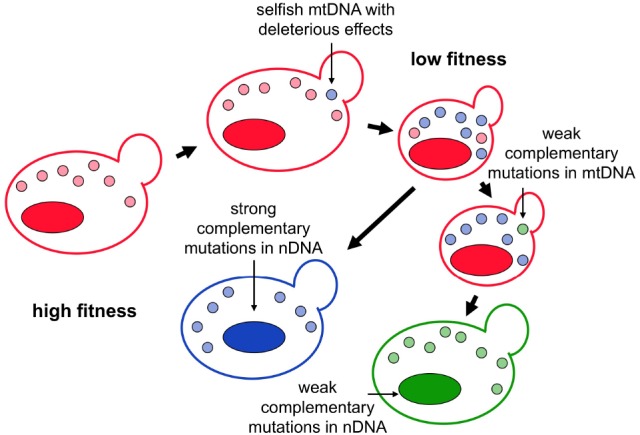
**The arms race and co-evolution between selfish mitochondrial DNA and the host cell.** Selfish mtDNA with a higher proliferation rate will gradually outcompete wild-type mtDNA in a sexual population. If the selfish mtDNA possesses harmful effects or carries other deleterious mutations, host fitness will be compromised after the selfish mtDNA reaches a certain frequency. When the selfish mtDNA is spreading through the population, the population starts to accumulate other mutations to restore the host fitness. Strong restoring mutations may occur in the nuclear genome that directly offset the harmful effect of the selfish mtDNA. Alternatively, weak restoring mutations may occur in both mitochondrial and nuclear genomes to compensate for the effect of deleterious mutations. In both scenarios, the evolved mitochondrial and nuclear genomes are different from the ancestral ones, but the host fitness remains the same.

The high fixation probability of mutations and frequent occurrence of selfish behavior in mitochondrial genomes suggest that the cooperative relationship between mitochondrial and nuclear genomes is unstable (Hurst, 1995; Sreedharan and Shpak, 2010; Hadjivasiliou et al., 2013; Jasmin and Zeyl, 2014). In an isolated population, the nuclear genome will be selected to counteract the deleterious effect caused by the selfish behavior. This antagonistic relationship is reminiscent of the arms race between parasites and their hosts, in which parasites constantly evolve new infectious strategies in order to spread and the hosts need to develop resistance to control parasite proliferation (Daugherty and Malik, 2012). The evolutionary arms race between nuclear and mitochondrial genomes can lead to rapid evolution of the genes involved in the interactions. It is worth noting that although mutations fixed in these two genomes are driven by adaptation (to increase the proliferation rate of mtDNA or nuclear genomes), the net outcome of the arms race may not be adaptive, meaning that the evolved cells have the same fitness as their ancestors. This co-evolution gives rise to what the “Red Queen” hypothesis predicts, that the nuclear genome has to run (evolve) hard in order to stay where it is (Figure 1; Van Valen, 1973).

In addition to the arms race, the nuclear genome may also be selected to compensate for the deleterious mutations that hitchhike with selfish mtDNA (leading to another type of compensatory coadaptation). The hitchhiking effect allows a population to fix rare deleterious mutations at a high rate (Fay and Wu, 2000). Moreover, unlike genetic drift, fixation of hitchhiking mutations is not heavily dependent on population size (Smith and Haigh, 1974). This type of compensatory coadaptation will lead to repeated rapid co-evolution between mitochondrial and nuclear genomes regardless of population size or the effect of mutations. Its evolutionary trajectory is more similar to intergenomic conflict but distinct from conventional compensatory coadaptation in which deleterious mitochondrial mutations are fixed by drift (Rice, 1987; Barton et al., 2013). Therefore, in later sections we will not separate it from intergenomic conflict in our discussion.

Rapid co-evolution between mitochondrial and nuclear genomes has been documented in many organisms. Osada and Akashi (2012) combined phylogenetic information and the 3D structure of the cytochrome c oxidase (*COX*) complex to reveal a strong tendency for co-evolution between mtDNA- and nuclear DNA (nDNA)-encoded components in primates. They found that rapid evolution in primate *COX* genes appears to be driven by adaptive evolution when nDNA-encoded mitochondrial proteins try to counteract deleterious nucleotide substitutions in mtDNA. In a systematic screen for cyto-nuclear incompatibility between closely related yeast species, our group found that an nDNA-encoded mitochondrial splicing protein, Mrs1, has co-evolved with the intron in the mtDNA-encoded *COX1* gene (Chou et al., 2010). They further confirmed that the functional change of Mrs1 is mainly caused by three amino acid changes localized on the RNA-binding surface.

The rapid co-evolution pattern is also observed in mitochondrial ribosomes, which are composed of proteins encoded in both mitochondrial and nuclear genomes. By comparing divergent copepod populations, Barreto and Burton (2012) found that the rate of amino acid changes for nuclear-encoded mitochondrial ribosomal proteins is higher than that of cytosolic ribosomal proteins. Similar patterns can be found at the interspecific level in wasps, flies and yeast. In plants, cyto-nuclear co-evolution has been examined using different ecotypes of *Arabidopsis thaliana*. Moison et al. (2010) found that in 27 pairs of the reciprocal F2 family, the cytoplasm donor has a significant effect on the germination capacity of seeds. Among these observed examples of fast co-evolution between mitochondrial and nuclear genomes, no direct evidence has shown that the driving mutations in mtDNA result from adaptation to environmental changes. Moreover, this phenomenon has been observed in a variety of organisms that have different population structures. It raises a doubt about whether they are all caused by conventional compensatory coadaptation in which population size plays a critical role. In the future, it will be important to examine the evolutionary trajectory of mitochondrial mutations and determine how often selfish mtDNA has been involved.

## The Red Queen and Hybrid Breakdown

**FIGURE 2:**
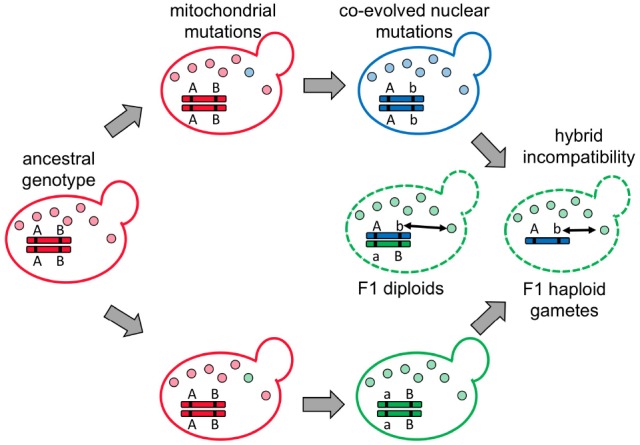
**Cyto-nuclear incompatibility represents a specific form of Dobzhansky-Muller incompatibility that causes hybrid breakdown.** In the ancestral population (in red), the nuclear genotype is AABB. When the population is split into two, different mitochondrial mutations (in blue and green) are fixed in each population. If the new mtDNA carries deleterious mutations (most likely due to hitchhiking with selfish elements) that compromise host fitness, the nuclear genomes may evolve complementary mutations (aa or bb) to restore the host fitness. When two populations containing co-adapted mitochondrial and nuclear mutations encounter each other and generate hybrids, unmatched mitochondria and nuclei (double-headed arrow) cause hybrid breakdown in F1 diploids (if the incompatibility is dominant), or F1 haploid gametes and F2 homozygous diploids (if the incompatibility is recessive).

Cyto-nuclear incompatibility is a specific form of Dobzhansky-Muller incompatibility, which is caused by improper interactions between genetic loci that have functionally diverged in two different species (Figure 2; Dobzhansky, 1937; Muller, 1942). As mentioned, many examples of cyto-nuclear incompatibility have been documented in a variety of organisms including primates (Kenyon and Moraes, 1997) and the details can be found in a very comprehensive recent review (Burton et al., 2013). A possible explanation for why cyto-nuclear incompatibility is so common among currently identified incompatible genes could be the arms race and co-evolution between mitochondrial and nuclear genomes. Unlike other speciation models, which suggest that the genes causing hybrid breakdown result from either adaptation to external ecological environments or genetic drift, the arms race model predicts that incompatibility can quickly evolve even if two populations are living in a constant environment similar to their ancestral populations (Johnson, 2010; Presgraves, 2010; Crespi and Nosil, 2013).

Hybrid incompatibility caused by internal evolutionary arms races has been suggested from studies in flies and plants (Fishman and Saunders, 2008; Bayes and Malik, 2009; Ferree and Barbash, 2009). However, the interactions between mitochondrial and nuclear genomes possess several unique features that are absent in other systems. First, mitochondria are the most important energy powerhouse of the cell, and the energy requirement usually is quite high during gametogenesis and sexual reproduction. Any reduction in the mitochondrial efficiency can easily compromise these processes. Second, all mtDNA-encoded genes need the assistance of nDNA-encoded proteins for their biogenesis or functions. This close relationship enables many mutations in mtDNA to drive co-evolution of nDNA-encoded mitochondrial proteins. Third, as we discussed previously, mtDNA is transmitted asexually, so any deleterious mutation in the same selfish mtDNA is unable to be separated when this selfish mtDNA is proliferating in the population. This implies that the same type of selfish elements may convey different deleterious effects when they occur in different populations. Cyclic arms races or co-evolution guarantee that cyto-nuclear incompatibility will frequently appear between different populations or species. However, most of our discoveries about cyto-nuclear incompatibility are from distinct species. Systematic studies among isolated populations are required to further validate the current model.

## Red Queen in Mitochondria and Human Disease

Despite the diminutive size of the mitochondrial genome, mtDNA mutations are an important cause of disease in human (Taylor and Turnbull, 2005; Tuppen et al., 2010). Mitochondrial biogenesis and function require regulated and coordinated expression of nuclear and mitochondrial genomes. Mutations in nDNA-encoded mitochondrial proteins can also lead to particular diseases associated with mitochondrial functions. Previously, it has been suggested that most population-specific mtDNA variations in modern humans are selectively neutral to avoid being eliminated by selection (Wallace et al., 1999). On the other hand, it remains elusive whether the neutrality is nuclear background-dependent. If arms races and co-evolution in mitochondrial and nuclear genomes have frequently happened in different human populations during evolution, the neutrality observed in a population may be specific to its nuclear background and an outcome of the balance between deleterious mtDNA mutations and nDNA-encoded suppressors. The deleterious effect of mtDNA variations will be revealed when they are introduced into a new nuclear background that does not contain the suppressors. Will the Red Queen Effect explain the complicated pattern in the diseases associated with mitochondrial defects? How many of those disease-related mitochondrial mutations are pathogenic only in certain nuclear backgrounds?

Many pathogenic mutations detected by genome-wide association studies (GWAS) also exist to a certain frequency among control populations (Manolio et al., 2009; Lee et al., 2011). Brandon et al. (2006) assembled and analyzed the data on somatic mtDNA mutations found in several types of tumors. They surprisingly discovered that among 190 tumor-specific somatic mtDNA mutations, 72% (137) of them overlap with mtDNA sequence variants in a human population database. These mutations cover a wide spectrum, including mutations in regulatory and protein-coding regions, tRNA, and rRNA. The biological relevance of the apparent association between tumor-specific mtDNA mutations and population variants is still unclear. However, it does support the opinion that mtDNA variants are not selectively neutral (Rand, 1994; Rand et al., 1994; Dowling et al., 2008). Recently, the development and use of human cybrids allow biologists to experimentally demonstrate the epistatic effect of mtDNA variants in different nuclear backgrounds (Lin et al., 2012; Kenney et al., 2014). These studies suggest that cyto-nuclear incompatibility can easily develop between different human populations. An even more important implication is that a mitochondria-related disease may simply be the outcome of mismatched mitochondrial and nuclear genomes.

## Can Compensatory Coadaptation Always Halt the Muller’s Ratchet?

Co-evolution of the nuclear genome allows cells to mitigate Muller’s ratchet in mitochondria. However, can the nuclear genome always keep up with its steps to maintain the host fitness when the mtDNA runs at different speeds? How will evolutionary trajectories of cyto-nuclear co-evolution change when mtDNA has different mutation rates or host cells have different population sizes? Since it is not easy to identify all footprints left by co-evolution between mitochondrial and nuclear genomes in natural populations, an alternative approach to these issues will be conducting experimental evolution in the laboratory and then analyzing the well-controlled evolved products. The baker’s yeast, *Saccharomyces cerevisiae* provides an excellent model organism for addressing these questions.

**FIGURE 3:**
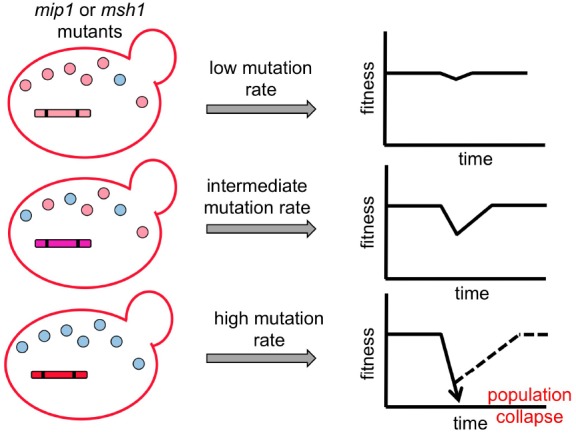
**Schematic models of co-evolution between selfish mtDNA and the nuclear genome.** Yeast cell lines with various mitochondrial mutation rates are constructed using different mutant forms of the mitochondrial DNA replication protein Mip1 and mismatch repair protein Msh1. Parallel sexual cultures are then set up to evolve in different population sizes. Outcrossed sex exposes mitochondrial genomes to competition and allows selfish mtDNA to spread despite organismal fitness costs. In addition, other deleterious mutations (shown as blue circles) have the opportunity to hitchhike with the selfish mtDNA when it is spreading through the population. When the frequency of deleterious mutations in mtDNA is increasing in the population, it results in a fitness valley. Subsequently, compensatory mutations in the nuclear genome will be selected to restore the host fitness unless the fitness valley is too deep to be recovered. This system allows us to assay the influence of different evolutionary parameters (i.e., mutation rate, population size and outcrossing frequency) on the co-evolution patterns between mitochondrial and nuclear genomes.

Different mutations in the yeast mtDNA polymerase Mip1, an ortholog of human pol γ that replicates mtDNA, can lead to 10- to 5000-fold increases in the mtDNA mutation rate (Hu et al., 1995; Ropp and Copeland, 1996; Baruffini et al., 2006). In addition, the mismatch repair system also plays a critical role in the fidelity of mtDNA replication (Chi and Kolodner, 1994). By manipulating both the mtDNA polymerase and mismatch repair proteins (such as Msh1), yeast cell lines with different levels of mtDNA mutation rates can be constructed and used to test co-evolution under a laboratory setup (Figure 3). This system will allow us to dissect the effect of mtDNA mutation rate and host cell population size on co-evolution of mitochondrial and nuclear genomes. Moreover, since mitochondrial or nuclear genomes can be easily separated in yeast, we can test the individual effect of evolved mtDNA and nuclear genomes by constructing cybrid cells carrying either evolved mitochondrial and ancestral nuclear genomes, or vice versa. Finally, the evolved genomes can be subjected to whole genome sequencing to identify the changes that occur during laboratory evolution. The combination of these analyses will generate a high-resolution map of how mitochondrial and nuclear genomes co-evolve within different parameters. The information can also help us understand the patterns observed in natural populations.

## Conclusion

It has been established that mitochondrial endosymbiosis played an important role in eukaryotic evolution. Using data from previous studies in a variety of organisms, we argue that mitochondria are also a strong driving force of population divergence. The arms race and co-evolution between mitochondrial and nuclear genomes allow different populations to accumulate different patterns of mutations, eventually leading to genetic incompatibility between populations. In some cases, the incompatibility may also contribute to human diseases despite that the cure already exists in the population. Cybrid line construction and the suppressor screen may provide a useful tool for deciphering the mitochondrial diseases. On the other hand, our understanding about the co-evolution patterns, evolutionary trajectories and determining parameters of this process is still very limited. Laboratory evolution experiments using a tractable organism like yeast may allow us to address these questions in a systematic manner.

### Conflict of Interest Statement

The authors declare that the research was conducted in the absence of any commercial or financial relationships that could be construed as a potential conflict of interest.
